# The Impact of COVID-19 on Mental Health Outcomes among Recipients of Ontario Social Assistance Benefits

**DOI:** 10.23889/ijpds.v9i5.2698

**Published:** 2024-09-18

**Authors:** Michael Campitelli, Eliane Kim, Lesley Plumptre, Astrid Guttmann, Paul Kurdyak

**Affiliations:** 1 ICES; 2 University of Toronto, Department of Paediatrics and Institute of Health Policy, Management and Evaluation; 3 The Hospital for Sick Children, Division of Paediatric Medicine and SickKids Research Institute; 4 Sunnybrook Research Institute; 5 University of Toronto, Department of Psychiatry; 6 The Centre for Addiction and Mental Health

## Objective and Approach

We linked the Ontario Social Assistance (OSA) Benefit Unit file from the Ministry of Children, Community and Social Services to provincial health administrative datasets housed at ICES to examine the impact of COVID-19 on Mental Health and Addictions (MHA) service use among OSA recipients.

Those receiving OSA benefits for February 2020 were matched to Ontario residents not receiving benefits based on age (±1 year), sex, income, expected resource utilization, and area of residence (N=771,891 matched pairs). We computed rates of MHA-related emergency department (ED) visits and hospitalizations in the 16-month period before (November 2018-February 2020) and after (March 2020-June 2021) pandemic onset.

## Results

MHA-related ED visit rates were much greater among OSA recipients (8.82 per 1,000 person-months) compared with matched controls (1.83 per 1,000 person-months) in the post-COVID period (Relative Rate [RR]=4.82; 95% Confidence Interval [95%CI] 4.75-4.89). Comparatively, MHA-related ED visit rates were also greater among OSA recipients (9.85 per 1,000 person-months) compared with controls (2.38 per 1,000 person-months) during the pre-COVID period (RR=4.13; 95%CI 4.08-4.18). The pre-COVID and post-COVID period RRs comparing OSA recipients and matched controls were significantly different (p<0.001). Results were consistent when examining MHA-related hospitalizations.

## Conclusions/Implications

While MHA-related ED visits and hospitalizations rates were significantly higher in OSA recipients than matched controls in both time periods, this difference increased following the onset of COVID-19. Increased reliance on hospital-based MHA services post-COVID represents the greater ongoing need for, and/or reduced access to, MHA-related ambulatory care for OSA recipients during the pandemic.

